# Effect of Tropisetron on Prevention of Emergence Delirium in Patients After Noncardiac Surgery: A Trial Protocol

**DOI:** 10.1001/jamanetworkopen.2020.13443

**Published:** 2020-10-14

**Authors:** Yi Sun, Dandan Lin, Jing Wang, Mengwen Geng, Mei Xue, Yayun Lang, Lina Cui, Yanan Hao, Shanshan Mu, Dan Wu, Lirong Liang, Anshi Wu

**Affiliations:** 1 Department of Anesthesiology, Beijing Chao-Yang Hospital, Capital Medical University, Beijing, China; 2 Department of Anesthesiology, Beijing Civil Aviation General Hospital, Beijing, China; 3 Department of Anesthesiology, Beijing Huairou District Hospital of Traditional Chinese Medicine, Beijing, China; 4 Department of Clinical Epidemiology and Tobacco Dependence Treatment Research, Beijing Chao-Yang Hospital, Capital Medical University, Beijing Institute of Respiratory Medicine, Beijing, China

## Abstract

**Importance:**

Postoperative delirium is a frequent disorder for patients undergoing surgery and is associated with poor outcomes. Delirium may occur in the immediate period after anesthesia administration and surgery. Tropisetron, which is frequently administrated for postoperative nausea and vomiting, is also a partial agonist of α7 nicotinic acetylcholine receptors associated with neuroprotective effects. Tropisetron may be the potential pharmacological treatment to decrease delirium after noncardiac surgery.

**Objective:**

To perform a randomized clinical trial to determine the efficacy and safety of tropisetron for prevention of emergence delirium in patients undergoing noncardiac surgery.

**Design, Setting, and Participants:**

This single-center, 2-arm randomized, double-blind, placebo-controlled trial will include 1508 patients undergoing noncardiac surgery. The intervention group will receive 5 mg of intravenous tropisetron before anesthesia induction, and patients in the control group will receive a placebo. The primary end point is the incidence of emergence delirium within 1 hour after tracheal tube removal, measured by the Confusion Assessment Method for the Intensive Care Unit score. The main secondary outcome is the incidence of postoperative delirium measured at 3 days of follow-up. An intention-to-treat principle will be used for all analyses.

**Discussion:**

Delirium remains the most common neuropsychiatric complication for patients after surgery. This will be the first randomized clinical study to evaluate whether tropisetron is effective in preventing emergence delirium. Results from this study will provide evidence for alteration of daily practice.

**Trial Registration:**

ClinicalTrials.gov Identifier: NCT04027751

## Key Points

**Question:**

Can tropisetron reduce the risk of emergence delirium in patients after noncardiac surgery?

**Findings:**

This trial protocol describes a randomized clinical trial of an estimated 1508 patients undergoing noncardiac surgery. The primary outcome is incidence of emergence delirium.

**Meaning:**

This ongoing clinical trial will supply evidence of the effectiveness of preoperative tropisetron treatment to reduce occurrence of emergence delirium.

## Teaser

This trial protocol describes a randomized clinical trial that aims to determine the efficacy and safety of tropisetron for prevention of emergence delirium in patients undergoing noncardiac surgery.

## Introduction

In patients undergoing anesthesia and surgery, approximately 11% to 51% will have postoperative delirium,^1^ which is characterized by cognitive decline, inattention, and a change in level of consciousness.^2^ It is a common neuropsychiatric complication of surgery, especially in elderly patients.^3^ Patients could present with delirium early in the postanesthesia care unit (PACU),^4,5,6^ with an incidence of 3.7% to 22.2%.^7,8,9^ The fluctuating mental status of delirium is associated with delayed postoperative recovery, longer hospital stay, and increased morbidity and mortality.^10,11,12^ Patients experiencing postoperative delirium are more likely to develop permanent cognitive disturbances.^13,14^ Thus, pharmacological prevention is needed to lower the risk of postoperative delirium and improve prognosis of patients.

Tropisetron is a serotonin (5-hydroxy-tryptamine) 3 receptor antagonist and is frequently used in prevention and treatment of postoperative nausea and vomiting.^15,16^ In addition, this drug is also a high-affinity partial agonist of α7 nicotinic acetylcholine receptors (α7-nAChRs).^17,18,19^ Triggering α7-nAChRs has demonstrated neuroprotective effects in various cognitive deficiency diseases.^20^ Most recently, beneficial effects in cognitive function, such as memory performance, have been reported of tropisetron in animals.^17,21^ In mouse models of Alzheimer disease, tropisetron induced greater improvements in spatial and working memory.^22^ The mechanism involves an increase in the ratio of α-secretase amyloid precursor protein to amyloid β_1-42_^18,22^ and protection against amyloid β-induced neurotoxicity.^23^ In a randomized double-blind study, patients with schizophrenia who received tropisetron were found to have overall improvement of cognitive deficits compared with patients who received a placebo.^24^ In perioperative settings, only a published hypothesis is available, showing that tropisetron could act as potential therapeutic drug for postoperative cognitive dysfunction, considering the shared identical mechanisms between postoperative cognitive dysfunction and Alzheimer disease.^25^ Taken together, evidence suggests that tropisetron is a promising approach in preventing delirium. However, no large clinical trials have studied the effect of tropisetron on emergence delirium. The aims of this study are (1) to determine the effect of tropisetron on the incidence of emergence delirium and (2) to better understand beneficial effects of tropisetron on the incidence of postoperative delirium and other delirium-related outcome measures compared with placebo.

## Methods

### Trial Status

The study is currently recruiting patients. Recruitment began August 1, 2019. We expect the study to be complete in December 2021. The trial protocol is available in the Supplement.

### Study Design and Setting

The study is being conducted in Beijing Chaoyang hospital affiliated with Capital Medical University, Beijing, China. The trial has been approved by the Medical Ethics Committee of the Chaoyang Hospital, Beijing. This study protocol is strictly in accordance with the Standard Protocol Items: Recommendations for Interventional Trials (SPIRIT) reporting guideline.^26,27^

The study is a double-blinded, randomized placebo-controlled trial that includes 1508 patients undergoing noncardiac surgery. Potential participants undergoing noncardiac surgery will be screened for eligibility criteria. All enrolled patients will be randomly divided into either the tropisetron group or placebo group at a 1:1 ratio. The primary end point is the incidence of emergence delirium.

### Eligibility Criteria

Inclusion criteria in the study are (1) 18 years or older; (2) general anesthesia for elective noncardiac surgery; (3) American Society of Anesthesiologists physical status score I to III; and (4) signed informed consent. Exclusion criteria are (1) history of neurological disease (eg, dementia or Parkinson disease); (2) neurosurgery; (3) history of psychiatric disease (eg, schizophrenia); (4) treatment with antipsychotic drugs during the last 30 days before enrollment; (5) unable to complete neuropsychological tests, including patients with severe visual or hearing impairment; (6) a Montreal Cognitive Assessment score of less than 10; (7) severe intraoperative adverse events (eg, cardiac arrest); and (8) contraindication to tropisetron.

### Recruitment and Consent

Participants are recruited in the hospital ward 1 day before surgery. Team members visit patients who meet the eligibility criteria and invite them to join the study. All participants sign the informed consent form before enrollment in this trial.

### Randomization and Blinding

Participants are randomly assigned to the tropisetron group or the placebo group with a 1:1 ratio with a block size of 4 (SAS software, version 9.4 [SAS Institute, Inc]). A statistician who is not involved in data collection or analysis (L.L.) produces the randomization list, which is printed out and sealed in an opaque envelope for each participant’s assignment. A study nurse (D.W.) assigns the participants to treatment by telephoning a contact in Beijing Chaoyang hospital. The contact is not involved in the number generation and recruitment process. Participants are then randomly allocated to identical ampoules with 5 mg of tropisetron or 0.9% saline solution. The syringe with a total volume of 1 mL is given to anesthesiologists before entering the operating room. Both participants and anesthesiologists are blinded to randomization assignments. Unblinding is permissible if necessary for safety reasons.

### Intervention and Study Visits

#### Baseline Hospital Ward Visit

Baseline assessments are conducted in the hospital ward. Demographic information, medical history, health history (eg, long-term alcohol use and smoking status), hearing and vision measurement, baseline laboratory tests, and electrocardiography are collected from the electronic medical record. One day before surgery, patients undergo assessments, including pain measured by a visual analog scale,^28,29^ depression by the 9-item Patient Health Questionnaire,^30^ anxiety by the 7-item Generalized Anxiety Disorder Scale,^31^ insomnia by Insomnia Severity Index,^32^ and cognitive function by the Montreal Cognitive Assessment,^33^ with the aim of identifying risk factors of emergence delirium. All planned study visits are presented in the Table.

**Table.** The Schedule of Planned Investigation

Study component	Visit 1	Visit 2	Visit 3	Visit 4	Visit 5	Visit 6	Visit 7	Visit 8
Time	1 d before surgery	Before anesthesia	During anesthesia	PACU	POD 1	POD 2	POD 3	Hospital discharge
Eligibility screen	X	−	−	−	−	−	−	−
Informed consent	X	−	−	−	−	−	−	−
Demographic characteristics	X	−	−	−	−	−	−	−
Randomization	X	−	−	−	−	−	−	−
Intervention	−	X	−	−	−	−	−	−
MoCA	X	−	−	−	−	−	−	−
VAS	X	−	−	X	X	X	X	−
PHQ-9	X	−	−	−	−	−	X	−
GAD-7	X	−	−	−	−	−	X	−
ISI	X	−	−	−	−	−	X	−
EEG frequency spectrum	−	X	X	X	−	−	−	−
Blood draw for biomarkers	−	X	−	X	−	−	−	−
CAM-ICU	−	−	−	X	X	X	X	−
Nausea and vomiting	−	−	−	X	X	X	X	−
Adverse events	−	−	−	X	X	X	X	−
Hospital stay	−	−	−	−	−	−	−	X

Abbreviations: CAM-ICU, Confusion Assessment Method for the Intensive Care Unit; EEG, electroencephalography; GAD-7, 7-item Generalized Anxiety Disorder Scale; ISI, Insomnia Severity Index; MoCA, the Montreal Cognitive Assessment; PHQ-9, 9-item Patient Health Questionnaire; POD, postoperative day; VAS, visual analog scale; X, present; minus sign, absent.

#### Baseline Operating Room Visit

An electroencephalography (EEG) monitor (the bispectral index [BIS; Covidien]) is applied in participants in both groups during the preoperative period (5 minutes with eyes closed). A blood sample is drawn before intervention for identifying potential biomarkers, including interleukin 1β (IL-1β), IL-6, IL-8, IL-10, IL-12p70, IL-17A, IL-18, IL-23, interferon γ, monocyte chemoattractant protein 1, receptor for advanced glycation end products, tumor necrosis factor, C-reactive protein, tau, phosphorylated tau 231, amyloid β_1-42_, vascular endothelial growth factor D, ubiquitin C-terminal hydrolase ligase-1, brain-derived neurotrophic factor, α-synuclein, and serum amyloid A.

### Intervention and Control

After randomization, patients enrolled in this trial come to the operating room and undergo standard monitoring, including electrocardiography, blood pressure, and oxygen saturation. Intravenous access is then established. The intervention group receives a 5-mg dosage of tropisetron as a bolus intravenously once before induction of anesthesia, whereas the control group receives placebo of 0.9% saline solution. Patients in both groups receive general anesthesia and tracheal intubation. Mean artery blood pressure is maintained above 65 mm Hg during surgery, and the bispectral value is maintained at 40 to 60. Electroencephalographic data are acquired by the bispectral monitoring system for the entire duration of the operation. Information is collected on surgical and anesthesia techniques used (eg, intraoperative medication, anesthesia methods, type of surgery, laparoscopic or open procedure, duration of surgery, estimated blood loss during surgery, intraoperative infusion, blood transfusion during surgery, and patient-controlled analgesia). Patients are transferred to the PACU after surgery. Patients in both groups receive nondrug interventions in the postoperative period. The Enhanced Recovery After Surgery pathway is not applied in our study.

### Follow-up Visits

In the PACU, participants are screened using the Confusion Assessment Method for the Intensive Care Unit (CAM-ICU)^34,35,36^ at 15 and 30 minutes after tracheal extubation and at discharge from PACU (within 1 hour after tracheal extubation). Delirium is defined as a positive CAM-ICU test result at either of these 3 time points. The Richmond Agitation Sedation Scale (RASS) is performed before the CAM-ICU test to assess the depth of sedation.^37^ If the RASS score is −4 or −5, the patient is insufficiently aroused for delirium assessment. Researchers will repeat an independent assessment at the next predetermined point. If the RASS score is greater than −4, the CAM-ICU test will be performed. If RASS scores remain −4 or −5 beyond 1 hour after tracheal extubation, the patient will no longer receive the CAM-ICU test in the PACU, and the primary outcome will be recorded as missing data. Before discharge from PACU, EEG monitoring (5 minutes) and blood draw are conducted for participants.

In the hospital wards, participants are reassessed for delirium from postoperative days 1 to 3 in the morning and the afternoon. The visual analog scale is used to measure the level of pain at postoperative days 1 to 3. Participants complete measures of depression (9-item Patient Health Questionnaire),^30^ anxiety (7-item Generalized Anxiety Disorder Scale),^31^ and insomnia (Insomnia Severity Index)^32^ at postoperative day 3. Trained researchers conduct assessments blinded to the allocation.

### Data Collection and Data Management

Data are collected through the medical record and patient visits. A study database with all included patients is generated, and the study-specific randomization module and variables from separate case report forms are entered into an electronic data set based on EpiData software, version 3.1 (EpiData Association). Consent and case report forms are locked in a research cabinet accessible to study researchers only and will be retained for 15 years after trial completion at Beijing Chaoyang Hospital. Quality control procedures are applied. All data will be merged at study end and exported to a database for further analysis.

### Outcomes

The primary outcome in this study is the incidence of emergence delirium within 1 hour after tracheal extubation measured using the validated Chinese version of the CAM-ICU.^36^ The CAM-ICU was validated against the *Diagnostic and Statistical Manual of Mental Disorders* (Fifth Edition) criteria for delirium in PACU settings based on the guideline.^38^ With a specificity of 98% and low sensitivity, we may detect the positive delirium cases conservatively in this study.^39^ The positive CAM-ICU test result requires the presence of (1) acute changes in mental status with a fluctuating course and (2) inattention, with either (3) disorganized thinking or (4) altered level of consciousness. Patients with delirium may display hyperactive or hypoactive signs. Hyperactive delirium is defined as RASS scores from 1 to 4, and hypoactive delirium is defined as RASS scores from −3 to 0.^40^ Secondary outcomes include (1) incidence of postoperative delirium within 3 days after surgery (defined as positive CAM-ICU test result); (2) nausea and vomiting within 3 days after surgery; (3) postoperative pain intensity at PACU discharge and 24, 48, and 72 hours after surgery, measured using the visual analog scale (scores range from 0-10, with higher scores indicating greater pain); (4) adverse events (cardiac arrhythmias, cardiogenic shock, and other unexpected adverse events) within 3 days after surgery; (5) length of hospital stay; and (6) all-cause mortality during hospitalization.

### Sample Size Calculation

The sample size calculation is based on the primary outcome. According to previous studies, the incidence of delirium diagnosed in the PACU ranged from 3.7% to 22.2%.^7,8,9^ We assumed an emergence delirium incidence of 15% in the control group and a 6% reduction (the clinical limit of superiority) in the intervention group. With a power of 80% and 1-sided α = .05 and using superiority tests for 2 proportions, 640 patients per group are required. With a dropout rate of 15%, the final planned total sample size is 754 randomized patients in each group. The sample size calculation was performed on PASS software, version 14.0 (Number Cruncher Statistical Software).

### Statistical Analysis

The primary analysis will be according to the intention-to-treat principle and complemented with a per protocol analysis.^41^ Nonadherence refers to patients who fail to receive the intervention or placebo after randomization, owing to cancelled operation or refusing to participate on the day of surgery. To control bias from nonadherence, sensitivity analyses will be conducted excluding participants with nonadherence.

### Baseline Analyses

To compare baseline characteristics, continuous variables will be given as mean (SD) for normal distribution or median (interquartile range) for skewed distribution. Categorical variables will be given as numbers (percentages). Clinical characteristics will be compared between the intervention and control groups using the unpaired 2-tailed *t* test or the Mann-Whitney test for continuous variables and with the use of the χ^2^ test or Fisher exact test for categorical variables.

### Primary Outcome Analysis

The primary outcome of our analysis is the incidence of emergence delirium. Comparisons in delirium incidence between the 2 groups will be assessed with a χ^2^ test, and 95% CIs will be calculated for the difference in delirium incidence.

### Priori Subgroup Analyses

Known confounders have an impact on delirium; thus, subgroup analyses will be conducted by comparisons of prespecified subgroups for the primary outcome. These include age (>65 years vs ≤65 years), surgery type (major vs minor), and preoperative Montreal Cognitive Assessment scores (>26 vs 18-26 vs 10-17) to eliminate the impact of these factors on tropisetron efficacy.

### Secondary Outcome Analysis

For secondary outcome analyses, the Mann-Whitney test will be used for length of hospital stay, whereas the χ^2^ test or the Fisher exact test will be used for categorical variables, including incidence of postoperative delirium within 3 days after surgery, nausea and vomiting, postoperative pain, and adverse events. For analysis of all-cause mortality during hospitalization, we will use Kaplan-Meier curves, with a log-rank test for between-group comparison. The Cox proportional hazards model will be used to estimate the hazard ratio among patients in the tropisetron group compared with the placebo group with adjusting for potential confounding factors.^42,43^

All statistical tests will be 2 sided, and statistical significance will be defined as *P* < .05. Statistical analysis will be performed with SPSS software, version 22.0 (SPSS, Inc).

### Prespecified Substudies

The primary purpose of this study is to determine whether tropisetron can lower the risk of emergence delirium and other delirium or treatment related outcomes. In addition, we will collect data on changes of blood biomarkers, EEG behaviors, and clinically relevant outcomes. Substudies derived from this trial are encouraged, with aim of providing more evidence on the following clinically relevant outcomes:

1. EEG behaviors and postoperative delirium. The role of EEG patterns for estimating and detecting delirium remains inconclusive.^44,45,46^ The present study will allow for this investigation, because EEG data are collected at baseline, the intraoperative period, and the postoperative period. We will explore the association between EEG behaviors and postoperative outcomes (eg, emergence delirium and postoperative delirium).
2. Association between blood biomarkers and postoperative delirium. Previous studies^47,48^ have evaluated the molecular levels of biomarkers associated with detection and/or estimating outcomes of postoperative delirium. In the present study, preoperative and postoperative blood samples were evaluated to explore for the association of potential biomarkers with the risk of postoperative delirium.
3. Effect of preoperative psychiatric and cognitive status on postoperative delirium. The cognitive status has an impact on development of postoperative delirium.^49,50^ We will explore the association between preoperative psychiatric and cognitive status (eg, anxiety, depression, insomnia, and cognitive impairment) and postoperative delirium.
4. Perioperative depressive symptoms outcomes. Depressive symptoms are a common and important complication of major surgery and associated with worse quality of life and even morality.^51^ Depressive symptoms at postoperative day 3 will be assessed and mechanisms will be explored, including risk factors, pain, and general health status.
5. Anxiety symptoms outcomes. The occurrence of anxiety has been shown to be associated with high levels of pain, prolonged hospital stays, and long-term dissatisfaction.^52^ Preoperative and postoperative anxiety symptoms will be assessed to determine clinical factors associated with presenting anxiety symptoms and postoperative outcomes (pain and general health status) associated with anxiety.
6. Association between insomnia and postoperative outcomes. Insomnia has previously been associated with clinically relevant outcomes.^53^ The study will assess the effect of preoperative insomnia on postoperative outcomes (pain and general health status).

### Safety and Adverse Event Reporting

The research team is monitoring the study for adverse events. Because the 5-mg dosage of tropisetron is commonly used to prevent nausea and vomiting in the perioperative period, serious adverse events attributable to tropisetron treatment are less likely to occur in the present study. The following adverse events will be reported: those possibly related to the study drug, such as headache, dizziness, diarrhea, and anaphylaxis, and serious events, including death and life-threatening events. Study oversight is performed by an independent data safety monitor board, composed of 2 physicians and a statistician. Interim analyses will be performed for safety concerns after including 500 and 1000 participants.

## Discussion

As far as we are aware, this is the first randomized clinical study powered to investigate tropisetron as a preventive drug for emergence delirium in adults scheduled for noncardiac surgery. At present, no effective drugs have been administrated in daily practice to prevent emergence delirium and subsequent cognitive and functional changes. Finding a treatment to decrease the risk of delirium and reduce overall discomfort in the immediate postoperative period is of great importance. The small body of existing evidence suggests that tropisetron could improve cognitive function by targeting α7 nAChR^20^; however, whether it would be effective in perioperative settings is unclear. To examine this question, we will use 5 mg of tropisetron before anesthesia induction as an intervention. It is pragmatic because this dosage of tropisetron is commonly applied to prevent nausea and vomiting during the perioperative period.

### Strengths and Limitations

There are several strengths of this study. The broad inclusion criteria of this study strengthens the external validity and clinical applicability. Predetermined subgroup analyses, such as age, surgery type, history of tobacco use, and other disturbances in cognitive function (anxiety, depression and insomnia) will mitigate these potential confounders, which could influence outcomes of our study.

This study has limitations that must be addressed. First, the trial is conducted at only 1 medical center, which restricts generalizability of the results. Second, symptoms of delirium fluctuate over time. Accurate identification of delirium at the assessing time may be difficult. We will perform delirium assessments at various times to ensure better detection.

## Conclusions

This randomized clinical trial will test the hypothesis that tropisetron could decrease the incidence of emergence delirium after noncardiac procedures in adults. The results will provide information about the efficacy and safety of tropisetron in preventing emergence delirium and lead to alterations in routine practice.

## Notes

Supplement. **Trial Protocol**
