# Supplementary material for: Effect of Tropisetron on Prevention of Emergence Delirium in Patients After Noncardiac Surgery: A Trial Protocol
Source: JAMA Netw Open. 2020 Oct 14;3(10):e2013443. doi: 10.1001/jamanetworkopen.2020.13443 (PMC7557499; doi:10.1001/jamanetworkopen.2020.13443)
Supplement: Supplement. — Trial Protocol [file jamanetwopen-e2013443-s001.pdf]

The Efficacy and Safety of Tropisetron in Preventing Emergence Delirium

Study Protocol

Principal Investigator

Anshi Wu, MD
Department of Anesthesiology
Bejijing Chao-Yang Hospital,
Capital Medical University
Telephone: 008610 85231330
Email: wuanshi1965@163.com

Key Abbreviations

BIS	the Bispectral Index
CAM-ICU	The Confusion Assessment Method for The Intensive Care Unit
CRF	case report form
DSMB	Data Safety Monitoring Board
ECG	electrocardiogram
EEG	Electroencephalography
GAD-7	7-item Generalized Anxiety Disorder Scale
ISI	Insomnia Severity Index
ITT	intention to treat analysis
MoCA	The Montreal Cognitive Assessment
NCT	National Clinical Trial
PACU	post anesthesia care unit
PHQ-9	9-item Patient Health Questionnaire
POCD	postoperative cognitive dysfunction
PONV	postoperative nausea and vomiting
PP	per protocol analysis
RASS	Richmond Agitation Sedation Scale
RCT	randomized controlled trial
VAS	Visual Analogue Scale

1 BACKGROUND

Postoperative delirium (POD) is a highly prevalent, costly and risky neuropsychiatric complication needed to be solved. In patients after anesthesia and surgery, approximately 11 to 51% of the population suffer from postoperative delirium,[1] which is characterized by cognitive decline, inattention and a change in level of consciousness.[2] It is a common neuropsychiatric complication of surgery, especially in the elderly.[3] Patients could present delirium early at post anesthesia care unit (PACU),[4-6] with an incidence of 3.7% to 22.2%.[7-9] Fluctuating mental status of delirium are associated with delayed postoperative recovery, longer hospital stay, increased morbidity and mortality.[10-12] Patients experienced postoperative delirium are more likely to develop permanent cognitivedisturbances.[13,14] Thus, pharmacological prevention is needed in order to lower the risk of postoperative delirium and improve prognosis of related patients.

The underlying molecular mechanisms of POD includes central cholinergic deficiency, neuroinflammation, oxidative stress and apoptosis. Acetylcholine plays an important role in regulating synaptic transmission and modulating cognitive performances.[15] Aging induces neurodegeneration, accompanied by decreased acetylcholine level and cholinergic dysfunction in cerebral cortex and hippocampus, and profoundly affects learning and memory processes.[16] The impaired cholinergic nervous system may predispose the patients to develop delirium.[17] Postoperative delirium may be caused by microglia activation, neuroinflammation and disruption of the blood-brain barrier (BBB).[1,18,19] Microglia activation triggers inflammatory cascade by release of proinflammatory cytokines, and results in the development of POD. [17] Accumulating evidence indicates that POD is correlated with elevated levels of multiple inflammatory cytokines such as IL-6, IL-2, TNF-a and IL-12.[20] Isoflurane exposure may induce neurotoxicity and increase risk of cognitive impairment for surgery patients.[21]

Activation of α7nicotinic acetylcholine receptor (α7nAChR) can inhibit inflammation, reduce the production of toxic Aβ-protein and lessen

neuronal apoptosis. α7nAChR is widely expressed in brain and its physiological functions have been demonstrated including cognition, sensation, analgesia, transmitter release and neurons protection.[22] Nicotine, choline and other α7nAChR agonist have been shown to attenuate inflammatory responses by reducing the release of pro-inflammatory cytokine.[23,24]

α7nAChR stimulation may activate signaling associated with neuroprotection which represent one approach for cognitive impairment treatment. α7nAChR bind $A\beta_{1-42}$ with high affinity, and $A\beta_{1-42}$ inhibits alpha7nAChR-dependent acetylcholine release which involves in cognitive functions.[25] Bitner suggested that α7nAChR agonist induced activation of phosphatidylinositol 3-kinase/ serine-threonine kinase (PI3K/AKT) pathway and showed a neuroprotective property by inhibition of glycogen synthase kinase 3β (GSK3β) activity and the concomitant reduction of tau phosphorylation attenuating tau hyperphosphorylation.[26] The protection effect of nicotine and galantamine against neurotoxicity has also been observed in the primary rat cortical neurons.[27] All these findings point out that α7nAChRs agonist may offers a potential approach for cognitive deficits.

As a partial agonist of α7nAchR, tropisetron is a feasible method that may participate in the regulation of cognitive function and provide neuroprotective benefits for POD in clinic. Tropisetron is a serotonin (5-hydroxy-tryptamine, 5-HT) 3 receptor antagonist and is frequently used in prevention and treatment of postoperative nausea and vomiting (PONV).[28,29] Additionally, this drug is also a high-affinity partial agonist of α7 nicotinic acetylcholine receptors (α7 nAChRs).[30-32] Triggering α7 nAChRhas been demonstrated neuroprotective effects in various cognitive deficiency diseases.[33] Most recently, beneficial effects in cognitive function such as memory performance have been reported of tropisetron in animals.[30,34] In Alzheimer's disease (AD) mouse models, tropisetroninduced greater improvements in spatial and working memory.[35] The mechanism involves increased the sAPPα/A β_{1-42} ratio[31,35] and protection against Aβ-induced neurotoxicity.[36] In a randomized double-blind study, schizophrenia patients who received tropisetron were found

to have overall cognitive deficits improved compared to patients received placebo.[37] In perioperative settings, only a published hypothesis is available, showing that tropisetron could act as potential therapeutic drug for postoperative cognitive dysfunction(POCD) considering the sharing identical mechanisms between POCD and Alzheimer's disease.[38] Taken together, evidence suggests that tropisetron comprises a promising approach in preventing delirium. However, there are no large clinical trials that have studied the effect of tropisetron for emergence delirium.

The aim of the study if to provide evidence on the effect of tropisetron in preventing delirium. This study based on a large sample, randomized, double-blind, placebo-controlled trial, combined scale evaluation with molecular biology measurement to investigate the efficacy and safety of POD prevention by tropisetron. The aim of this study is firstly to determine the effect of tropisetron on the incidence of emergence delirium. Secondly, to better understand beneficial effects of tropisetron on the incidence of postoperative delirium and other delirium related outcome measures compared with placebo. Once tropisetron was proved can reduce the incidence of POD, we will be pioneered in provide a new approach for preventing POD, which has important economic and social benefits for reducing postoperative complications and improving the prognosis of patients in the future.

2 HYPOTHESIS AND STUDY OBJECTIVE

2.1 Hypothesis

Tropisetron will lead to lower risk of emergence delirium for patients undergoing non-cardiac surgery.

2.2. Study Objective

To perform a randomized clinical trial with aim of determining the efficacy and safety of tropisetron on prevention of emergence delirium in patients undergoing non-cardiac surgery.

3 STUDY METHODS

3.1 Overall Study Design

We will conduct a double-blinded, randomized placebo-controlled trial that includes 1508 patients undergoing non-cardiac surgery. Potential participants undergoing non-cardiac surgery will be screened for eligible criteria and sign the informed consent form before recruitment in this trial. All enrolled patients will be randomly divided into either Tropisetron group or placebo group at a 1:1 ratio. The patients will receive a screening visit, a pre-intervention visit, an intraoperative visit and five follow-up visits. The primary endpoint is the incidence of emergence delirium. We will also examine important outcomes such as incidence of postoperative delirium within 3 days after surgery, nausea and vomiting, postoperative pain, adverse events, and length of hospital stay.

3.2 Participant selection

3.2.1 Inclusion Criteria

- Age 18 years or older
- Written consent given
- Scheduled to undergo elective non-cardiac surgeries under general anesthesia
- American Society of Anesthesiologists physical status score I-III

3.2.2 Exclusion Criteria

- History of neurological disease (e.g., dementia, or Parkinson’s disease)
- Patients undergoing neurosurgery
- History of psychiatric disease (e.g., schizophrenia)
- Patients with a medication history of anti-psychiatric drugs over the last 30 days prior to enrollment
- Unable to complete neuropsychological tests including patients with severe visual or hearing impairment
- The Montreal Cognitive Assessment (MoCA) scores below 10

- Patients with severe intraoperative adverse events (e.g., cardiac arrest)
- Patients with contraindication to tropisetron.

3.3 Recruitment

Participants are recruited in hospital ward one day before surgery at Beijing Chao-Yang Hospital. Team members will screen and visit patients who meet the eligibility criteria and invite them to join the study.

3.4 Informed Consent

All participants signed written informed consent forms before enrollment. During the consent process, investigators will: (1) give an introduction about the study; (2) explain the trial in detail including all collected data; (3) inform of benefits and risks during trial procedure; (4) explain the nondisclosure agreements with participants; (5) answer participants' questions; (6) given a contact information for study investigator. Consent forms will be locked in a research cabinet.

3.5 Randomization and Blinding

Participants will be randomly assigned to tropisetron group or placebo group with a 1:1 ratio with a block size of 4 (SAS software, version 9.4， SAS institute Inc, USA). A statistician who is not involved in data collection or analysis produced the randomization list which is printed out and sealed in an opaque envelope for each participant's assignment. A study nurse (Dan Wu) who is blinded to the participants' characteristics assigned the participants to treatment by telephoning a contact in Beijing Chao-Yang hospital. The contact is not involved in the number generation and recruitment process. Participants will be then randomly allocated to identical ampoules with 5 mg tropisetron or 0.9% saline solution. The syringe with a total volume of 1 mL will be given to anesthesiologists prior to entering the operating room. Both participants and anesthesiologists are blinded to randomization assignments. Unblinding is permissible if necessary for safety reasons.

3.6 Intervention and Study Visits

3.6.1 Baseline Hospital Ward Visit

Baseline assessments are conducted in the hospital ward. Demographic information, medical history, health history (e.g., chronic alcohol use and smoking status), hearing and vision condition, baseline laboratory tests and electrocardiogram are collected from electronic medical record. Participants receive assessments, including pain measured by Visual Analogue Scale (VAS; pain assessment),[39,40] depression by 9-item Patient Health Questionnaire (PHQ-9; depression assessment),[41] anxiety by 7-item Generalized Anxiety Disorder Scale (GAD-7),[42] insomnia by Insomnia Severity Index (ISI)[43] and cognitive functions by the Montreal Cognitive Assessment (MoCA),[44] with aim of identifying risk factors of emergence delirium.

3.6.2 Baseline Operating Room Visit

Electroencephalography (EEG) monitor (the Bispectral Index, BIS) is applied in participants in both groups during preoperative period (5min with eyes closed). A blood sample is drawn prior to intervention for identifying potential biomarkers including IL-1β, IL-6, IL-8, IL-10, IL-12p70, IL-17A, IL-18, IL-23, IFN-γ, MCP-1, RAGE, TNF-α, CRP, Tau, Tau[pT231], Aβ1-42, VEGF-D, UCHL1, BDNF, α-Synuclein, SAA.

3.6.3 Intervention and Control

Following randomization, patients enrolled in this trial come to the operating room and are applied with standard monitoring, including electrocardiogram (ECG), blood pressure and oxygen saturation. Intravenous access is then established. The intervention group receives tropisetron in a dosage of 5 mg as a bolus intravenously once prior to induction of anesthesia, while the control group receives placebo of 0.9% saline solution. Patients in both groups receive general anesthesia and tracheal intubation. Mean artery blood pressure (MAP) is maintained more than 65 mmHg during surgery, BIS value is maintained between 40 and 60. EEG data is acquired by the BIS monitoring system for the

entire duration of the operation. Information is collected on surgical and anesthesia techniques used (e.g., intraoperative medication, anesthesia methods, type of surgery, laparoscopic or open, duration of surgery, estimated blood loss during surgery, intraoperative infusion, blood transfusion during surgery and patient-controlled analgesia). Patients are transferred to post-anesthesia care unit (PACU) after surgery. Patients of both group receive non-drug intervention in the postoperative period. The Enhanced Recovery After Surgery (ERAS) pathway is not applied in our study.

3.6.4 Follow-up Visits

In the PACU, participants will be screened by The Confusion Assessment Method for The Intensive Care Unit (CAM-ICU)[45-47] at 15mins, 30mins after tracheal extubation, and at discharge from PACU (within 1 hour after tracheal extubation). Delirium will be defined as a positive CAM-ICU test at either of these three time points. Richmond Agitation Sedation Scale (RASS) will be performed before CAM-ICU test in order to assess depth of sedation.[48] If RASS scores is -4 or -5, the patient is insufficiently aroused for delirium assessment. Researchers will repeat an independent assessment at next predetermined time point. If RASS scores is >-4, the patients will receive following CAM-ICU test. If RASS scores remain -4 or -5 beyond 1 hour after tracheal extubation, the patient will no longer receive CAM-ICU test at PACU, and the primary outcome will be recorded as missing data.

In the hospital wards, participants are reassessed for delirium from postoperative day one to day 3, twice a day, in the morning and afternoon. Visual Analogue Scale is performed to measure level of postoperative pain at postoperative day 1-3. Participants will complete measures of depression (9-item Patient Health Questionnaire, PHQ-9),[41] anxiety (7-item Generalized Anxiety Disorder Scale, GAD-7)[42] and insomnia (Insomnia Severity Index, ISI)[43] at postoperative day 3. Trained researchers will conduct assessment blinded to the allocation.

3.7 Data Collection and Management·

3.7.1 Outcome Measurements

Montreal cognitive assessment (MoCA). The MoCA will be used to evaluate cognitive function. The MoCA is a multi-dimension cognitive assessment that evaluate visuospatial, executive, naming, memory, attention, language, abstraction, delayed recall and orientation. The total score is 30 and score lower than 26 indicates cognitive impairment. MoCA has comparable sensitivity and specificity to diagnose mild cognitive impairment.[44]

Richmond agitation-sedation scale (RASS). The RASS is a reliable and valid sedation scale which will be used to detect changes in sedation status. It has 10 scale representing discrete criteria for different levels of sedation and agitation. And the scale is measured according to duration of eye contact aroused by stimulation (verbal or physical).[49]

Confusion assessment method intensive care (CAM-ICU). The CAM-ICU is a reliable assessment with high level of sensitivity and specificity, and will be used to measure delirium. It is valid for both verbal and nonverbal patients. The contents focus on acute onset of mental status changes or fluctuating course, inattention, disorganized thinking and altered level of consciousness.[50]

9-item patient health questionnaire (PHQ-9). The PHQ-9 will be used to measure depressive disorder and the grade of depressive symptom severity. It is completely self-administered and has been commonly used in clinical evaluation. The total score range from 0 to 27, with 0 to 3 points for each item. The thresholds for mild, moderate, moderately severe and severe depression are 5, 10, 15 and 20.[41]

7-item generalized anxiety disorder (GAD-7). The GAD-7 will be used to screen anxiety and measure its severity. The participant rates each item using scale from 0 (not at all) to 3 (nearly every day). And the degree of anxiety which is defined as minimal, mild, moderate and severe is based on the total score for 7 items.[51]

Insomnia severity index (ISI). The ISI will be used to assess subjective insomnia. It

is a self-report instrument measuring insomnia symptoms and consequences. The items on the were designed to assess the severity of sleep-onset, sleep maintenance difficulties, satisfaction with current sleep pattern, interference with daily functioning, notice ability impairment and degree of distress or concern caused by sleeping problems. [52]

Visual analogue score (VAS). The VAS is an easy and reliable assessment that will be used to measure the intensity of pain. A ruler is provided, which has picture of faces showing none to extremely pain on one side and continuous scale on the reverse side. The participant will be asked to point out the status of pain and the corresponding score will be acquired. The pain intensity can be categorized as none, mild, moderate and severe.[53]

3.7.2 EEG Data Acquisition and Processing

EEG data is acquired by the BIS monitoring system for the entire duration of the operation and recorded whether or not burst suppression is present. The baseline eyes closed period (5 min), and the recovery period after extubation (5 min) are extracted from the data for each patient.

Data is reviewed in EEGLab to assess for quality and the presence of motion and eye movement artifact. Spectral analysis is performed with Chronux Toolbox and MATLAB (MathWorks, USA) scripts. Spectral power is computed with multitaper spectral analyses (mtspecgramc function; time window: 6 s, overlap: 0 s, number of tapers: 3, time-bandwidth product: 5, spectral resolution: 0.25 Hz). The median absolute power (10*log10[μV2/Hz]) is calculated for the recovery period at each of three frequency bands (delta: 1 to 4 Hz, theta: 4 to 8 Hz, alpha: 8 to 13 Hz) for frontal channels.

3.7.3 Blood Biomarker

Intravenous blood samples are collected in 5ml tube, centrifuged at 4,000×g for 10 min. Supernatants are stored at −80 °C prior to the determination of IL-1β, IL-6, IL-8, IL-10, IL-12p70, IL-17A, IL-18, IL-23, IFN-γ, MCP-1, RAGE, TNF-α, CRP, Tau, Tau[pT231], Aβ1-42, VEGF-D, UCHL1, BDNF, α-Synuclein, SAA.

3.7.4 Demographic Assessments

Demographics. Baseline assessments will be conducted in preoperative visit in hospital wards. The information collected include age, gender, BMI, education status, living status, ethnic, history of physical diseases, smoking and alcohol consumption. And laboratory testes such as blood routine, blood biochemistry, blood pressure and electrocardiogram will be obtained from electronic medical record.

Surgery and anesthesia information. The information of surgery and anesthesia including the grade of ASA, anesthesia methods, intraoperative anesthetic, type of surgery, laparoscopic or open, duration of surgery, estimated blood loss during surgery, intraoperative infusion, blood transfusion during surgery and patient-controlled analgesia. All the information will be acquired from electronic medical record.

Follow-up information. Participants will be asked about VAS and postoperative nausea and vomiting for 3 days after surgery. If the participant suffers from unbearable postoperative pain, additional analgesia such as tramadol and flubiprofen will be used for pain management. Name and dosage of additional analgesia will be recorded. The adverse events such as shiver, hypertension, hypotension, hypoxia, major adverse cardiac events, atelectasis, pulmonary edema, acute kidney injury, death are also screened for 3 days.

3.7.4 Data Management

A study database with all included patients will be generated, and the study specific randomization module and study specific variables from separate case report forms (CRFs) will be entered into an electronic dataset based on EpiData software. Consent forms and CRFs will be locked in a research cabinet accessible to study researchers only, and will be retained for 15 years after trail completion at Beijing Chao-Yang Hospital. Quality control procedures will apply. All data will be merged at study end and exported to an analysis database for further analysis.

3.8 Outcomes

3.8.1 Primary Outcome

The primary outcome in this study is the incidence of emergence delirium within 1h after tracheal extubation measure using the validated Chinese version of the CAM-ICU.[47] The positive CAM-ICU test requires the presence of (1) acute changes in mental status of a fluctuating course and (2) inattention, with either (3) disorganized thinking or (4) altered level of consciousness. Patients with delirium may display hyperactive signs or hypoactive signs. Hyperactive delirium is defined as RASS scores between +1 and +4, and hypoactive delirium is defined as RASS scores between -3 and 0.[54]

3.8.2 Secondary Outcomes

Secondary outcomes include: (1) incidence of postoperative delirium within 3 days after surgery (defined as positive CAM-ICU test); (2) nausea and vomiting; (3) postoperative pain, measured using VAS (Score ranges from 0 to 10, with higher scores indicating greater pain); (4) adverse events (cardiac arrhythmias, cardiogenic shock and other unexpected adverse events); (5) length of hospital stay; (6) all-cause mortality

3.9 Sample Size Calculation

The sample size calculation is based on the primary outcome. According to previous studies, incidence of delirium diagnosed in the PACU ranged between 3.7% and 22.2%.[7-9] We assumed emergence delirium incidence of 15% in the control and a 6% reduction (the clinical limit of superiority) in the intervention group. With a power of 80% and one-sided alpha level of 0.05, using superiority tests for two proportions, 640 patients per group are required. Considering a dropout rate of 15%, the final planned total sample size is 754 randomized patients in each group. The sample size calculation was performed on PASS 14.0 Software (Number Cruncher Statistical Software, USA).

4 STATISTICAL ANALYSES

4.1 Intention to Treat Analysis

The primary analysis will be according to the intention to treat (ITT) principle and complemented with a per protocol (PP) analysis.[55] Non-compliance refers that patients fail to receive intervention or placebo after randomization, such as cancelled operation and refusing to participate on surgery day. To control bias from non-compliance, sensitivity analyses will be conducted among participants without non-compliance.

4.2 Baseline Analyses

To comparing baseline characteristics, continuous variables will be given as mean (standard deviation [SD]) for normal distribution or median (interquartile range [IQR]) for skewed distribution. Categorical variables will be given as numbers and percentages. Clinical characteristics were compared between the two groups of patients with the use of an analysis of Student's t-test or the Mann–Whitney U test for continuous variables and with the use of the $\chi 2$ test or Fisher exact test for categorical variables.

4.3 Primary Outcome Analysis

The primary outcome of our analysis is the incidence of emergence delirium. Comparisons in delirium incidence between two groups will be assessed with a $\chi 2$ test, and 95% CI will be calculated for the difference in delirium incidence.

4.3.1 *Priori* Subgroup Analyses

To explore the results differences amongst different patients, subgroup analyses will be conducted by comparisons of prespecified subgroups: a. Age (older than 65 years versus 65 years or younger); b. Surgery type (major surgery versus minor surgery); c. preoperative MoCA scores (>26 versus 18-26 versus 10-17).

4.4 Secondary Outcome Analysis

For secondary outcome analyses, the Mann–Whitney U test will be used for length of hospital stay, while $\chi 2$ test or Fisher exact test will be used for

categorical variables, including incidence of postoperative delirium within 3 days after surgery, nausea and vomiting, postoperative pain, adverse events. For all-cause mortality during hospitalization, we will conduct Kaplan-Meier curves, with a log-rank test for between-group comparison. Cox proportional hazard model will be used to estimate the hazards ratio among patients in tropisetron group compared with those with placebo with adjusting for potential confounding factors.[56,57]

All tests will be two-sided and statistical significance will be defined as a P value less than 0.05. Statistical analysis will be performed with SPSS 22.0 software (SPSS Inc, USA).

5 PRESPECCIFIED SUB-STUDIES

The primary purpose of this study is to determine whether tropisetron can lower the risk of emergence delirium and other delirium or treatment related outcomes. In addition, we collect data on changes of blood biomarkers, EEG behaviors and clinically relevant outcomes. Sub-studies derived from this trial are encouraged, with aim of providing more evidence on clinically relevant outcomes as below:

a. EEG behaviors and postoperative delirium

It remains inconclusive on the role of EEG patterns for delirium prediction and detection.[58-60] The current study allows for this investigation, as EEG data is collected on baseline, intraoperative period and postoperative period. We will explore the relationship between EEG behaviors and postoperative outcomes (e.g., emergence delirium and postoperative delirium).

b. Association between blood biomarkers and postoperative delirium

Previous study has evaluated the molecular levels of biomarkers related to detection and/or predicting prognosis of postoperative delirium.[20,61] In the current study, preoperative and postoperative blood samples were measured

to explore for the association of potential biomarkers with the risk of postoperative delirium.

c. Effect of preoperative psychiatric and cognitive status on postoperative delirium

The cognitive status has impact on development of postoperative delirium.[62,63] We will explore the association between preoperative psychiatric and cognitive status (e.g. anxiety, depression, insomnia and cognitive impairment) and postoperative delirium.

d. Perioperative depressive symptoms outcomes

Depressive symptoms are a common and important complication of major surgery and associated with worse quality of life and even morality.[64] Depressive symptoms at postoperative day 3 will be assessed and mechanisms would be explored including: risk factors, pain and general health status

e. Anxiety symptoms outcomes

The occurrence of anxiety has been shown to be associated with high levels of pain, prolonged hospital stays and long-term dissatisfaction.[65] Preoperative and postoperative anxiety symptoms will be assessed to determine clinical factors associated with presenting anxiety symptoms, and postoperative outcomes (pain and general health status) associated with anxiety.

f. Association between insomnia and postoperative outcomes

Insomnia have previously been associated with clinically relevant outcomes.[66] The study will assess the effect of preoperative insomnia on postoperative outcomes (pain and general health status).

6 SAFETY AND ADVERSE EVENT REPORTING

The research team is monitoring the study for adverse events. Since tropisetron 5mg is commonly used to prevent PONV in perioperative period, it would be less likely that serious adverse events attributable to tropisetron treatment would occur in the current study. The following adverse events will be reported: adverse events possibly related to the study drug, such as headache, dizziness, diarrhea and anaphylaxis, and serious adverse event including death and life-threatening. Study oversight is performed by an independent Data Safety Monitor Board (DSMB), which is composed of two physicians and a statistician. Interim analyses will be performed for safety concerns after including 500, 1000 participants respectively.

7 STRENTHS AND LIMITATIONS

As far as we are aware, this is the first randomized controlled study powered to investigate tropisetron as a prevention drug for emergence delirium in adults scheduled for non-cardiac surgery.

There are several strengths of this study. The broad inclusion of this study strengthens the external validity and clinical applicability. Predetermined subgroup analysis, such as age, surgery type, smoke history and other disturbance in cognitive function (anxiety, depression and insomnia) will mitigate these potential confounders which could influence outcomes of our study.

This study has limitations which must be addressed. First, the trial is conducted at only one medical center, which restricts generalizability of the results. Second, symptoms of delirium fluctuate over times. It may be difficult to accurately identify delirium at the assessing time. We will perform delirium assessments at various times to ensure better detection.

8 ETHNIC

This protocol will be reviewed and approved by the Medical Ethics Committee of the Chaoyang Hospital. This protocol will be registered in Clinicaltrials.gov. All study members involved in the conduct of this research will receive the required education on the protection of human participant rights.

REFERENCES

1. Inouye SK, Westendorp RG, Saczynski JS. Delirium in elderly people. Lancet 2014;383:911-22.
2. Inouye SK, Westendorp RG, Saczynski JS. Delirium in elderly people. Lancet;383:911-22.
3. Evered L, Silbert B, Knopman DS et al. Recommendations for the Nomenclature of Cognitive Change Associated with Anaesthesia and Surgery-2018. Anesthesiology 2018;129:872-9.
4. Hernandez BA, Lindroth H, Rowley P et al. Post-anaesthesia care unit delirium: incidence, risk factors and associated adverse outcomes. Br J Anaesth 2017;119:288-90.
5. Fields A, Huang J, Schroeder D, Sprung J, Weingarten T. Agitation in adults in the post-anaesthesia care unit after general anaesthesia. Br J Anaesth 2018;121:1052-8.
6. Neufeld KJ, Leoutsakos JM, Sieber FE et al. Outcomes of early delirium diagnosis after general anesthesia in the elderly. Anesth Analg 2013;117:471-8.
7. Munk L, Andersen G, Moller AM. Post-anaesthetic emergence delirium in adults: incidence, predictors and consequences. Acta Anaesthesiol Scand 2016;60:1059-66.
8. Card E, Pandharipande P, Tomes C et al. Emergence from general anaesthesia and evolution of delirium signs in the post-anaesthesia care unit. Br J Anaesth 2015;115:411-7.
9. Kim HJ, Kim DK, Kim HY, Kim JK, Choi SW. Risk factors of emergence agitation in adults undergoing general anesthesia for nasal surgery. Clin Exp Otorhinolaryngol 2015;8:46-51.
10. Marcantonio ER. Postoperative delirium: a 76-year-old woman with delirium following surgery. JAMA 2012;308:73-81.
11. Whitlock EL, Torres BA, Lin N et al. Postoperative delirium in a substudy of cardiothoracic surgical patients in the BAG-RECALL clinical trial. Anesthesia and analgesia 2014;118:809-17.

12. Moskowitz EE, Overbey DM, Jones TS et al. Post-operative delirium is associated with increased 5-year mortality. Am J Surg 2017;214:1036-8.
13. Shi Z, Mei X, Li C et al. Postoperative Delirium Is Associated with Long-term Decline in Activities of Daily Living. Anesthesiology 2019.
14. Rudolph JL, Marcantonio ER, Culley DJ et al. Delirium is associated with early postoperative cognitive dysfunction. Anaesthesia 2008;63:941-7.
15. Schliebs R, Arendt T. The significance of the cholinergic system in the brain during aging and in Alzheimer's disease. J Neural Transm (Vienna) 2006;113:1625-44.
16. Schliebs R, Arendt T. The cholinergic system in aging and neuronal degeneration. Behav Brain Res 2011;221:555-63.
17. van Gool WA, van de Beek D, Eikelenboom P. Systemic infection and delirium: when cytokines and acetylcholine collide. Lancet 2010;375:773-5.
18. Hu N, Guo D, Wang H et al. Involvement of the blood-brain barrier opening in cognitive decline in aged rats following orthopedic surgery and high concentration of sevoflurane inhalation. Brain Res 2014;1551:13-24.
19. Cao Y, Ni C, Li Z et al. Isoflurane anesthesia results in reversible ultrastructure and occludin tight junction protein expression changes in hippocampal blood-brain barrier in aged rats. Neurosci Lett 2015;587:51-6.
20. Androsova G, Krause R, Winterer G, Schneider R. Biomarkers of postoperative delirium and cognitive dysfunction. Front Aging Neurosci 2015;7:112.
21. Zhang B, Tian M, Zhen Y et al. The effects of isoflurane and desflurane on cognitive function in humans. Anesth Analg 2012;114:410-5.
22. Gotti C, Zoli M, Clementi F. Brain nicotinic acetylcholine receptors: native subtypes and their relevance. Trends in pharmacological sciences 2006;27:482-91.
23. Liu Y, Yang J, Bao J et al. Activation of the cholinergic anti-inflammatory pathway by nicotine ameliorates lipopolysaccharide-induced preeclampsia-like symptoms in pregnant rats. Placenta 2017;49:23-32.
24. Matsuda A, Jacob A, Wu R et al. Novel therapeutic targets for sepsis: regulation of exaggerated inflammatory responses. Journal of Nippon

Medical School = Nippon Ika Daigaku zasshi 2012;79:4-18.
25. Wang HY, Lee DH, D'Andrea MR, Peterson PA, Shank RP, Reitz AB. beta-Amyloid(1-42) binds to alpha7 nicotinic acetylcholine receptor with high affinity. Implications for Alzheimer's disease pathology. The Journal of biological chemistry 2000;275:5626-32.
26. Bitner RS, Nikkel AL, Markosyan S, Otte S, Puttfarcken P, Gopalakrishnan M. Selective alpha7 nicotinic acetylcholine receptor activation regulates glycogen synthase kinase3beta and decreases tau phosphorylation in vivo. Brain research 2009;1265:65-74.
27. Kihara T, Sawada H, Nakamizo T et al. Galantamine modulates nicotinic receptor and blocks Abeta-enhanced glutamate toxicity. Biochemical and biophysical research communications 2004;325:976-82.
28. Kovac AL. Prevention and treatment of postoperative nausea and vomiting. Drugs 2000;59:213-43.
29. Tricco AC, Soobiah C, Blondal E et al. Comparative safety of serotonin (5-HT3) receptor antagonists in patients undergoing surgery: a systematic review and network meta-analysis. BMC Med 2015;13:142.
30. Callahan PM, Bertrand D, Bertrand S, Plagenhoef MR, Terry AV, Jr. Tropisetron sensitizes alpha7 containing nicotinic receptors to low levels of acetylcholine in vitro and improves memory-related task performance in young and aged animals. Neuropharmacology 2017;117:422-33.
31. Hashimoto K. Tropisetron and its targets in Alzheimer's disease. Expert Opin Ther Targets 2015;19:1-5.
32. Zulkifli MH, Viswenaden P, Jasamai M, Azmi N, Yaakob NS. Potential roles of 5-HT3 receptor (5-HT3R) antagonists in modulating the effects of nicotine. Biomed Pharmacother 2019;112:108630.
33. Bertrand D, Lee CH, Flood D, Marger F, Donnelly-Roberts D. Therapeutic Potential of alpha7 Nicotinic Acetylcholine Receptors. Pharmacol Rev 2015;67:1025-73.
34. Poddar I, Callahan PM, Hernandez CM, Yang X, Bartlett MG, Terry AV, Jr. Tropisetron enhances recognition memory in rats chronically treated with risperidone or quetiapine. Biochem Pharmacol 2018;151:180-7.
35. Spilman P, Descamps O, Gorostiza O et al. The multi-functional drug

tropisetron binds APP and normalizes cognition in a murine Alzheimer's model. Brain Res 2014;1551:25-44.

36. Rahimian R, Fakhfouri G, Ejtemaei Mehr S et al. Tropisetron attenuates amyloid-beta-induced inflammatory and apoptotic responses in rats. Eur J Clin Invest 2013;43:1039-51.

37. Zhang XY, Liu L, Liu S et al. Short-term tropisetron treatment and cognitive and P50 auditory gating deficits in schizophrenia. Am J Psychiatry 2012;169:974-81.

38. Yang C, Ren Q, Zhang JC, Hashimoto K. Tropisetron for postoperative cognitive decline. Aust N Z J Psychiatry 2015;49:662-3.

39. Collins SL, Moore RA, McQuay HJ. The visual analogue pain intensity scale: what is moderate pain in millimetres? Pain 1997;72:95-7.

40. Zusman M. The Absolute Visual Analogue Scale (AVAS) as a Measure of Pain Intensity. Aust J Physiother 1986;32:244-6.

41. Kroenke K, Spitzer RL, Williams JB. The PHQ-9: validity of a brief depression severity measure. J Gen Intern Med 2001;16:606-13.

42. Lowe B, Decker O, Muller S et al. Validation and standardization of the Generalized Anxiety Disorder Screener (GAD-7) in the general population. Med Care 2008;46:266-74.

43. Morin CM, Belleville G, Belanger L, Ivers H. The Insomnia Severity Index: psychometric indicators to detect insomnia cases and evaluate treatment response. Sleep 2011;34:601-8.

44. Nasreddine ZS, Phillips NA, Bedirian V et al. The Montreal Cognitive Assessment, MoCA: a brief screening tool for mild cognitive impairment. J Am Geriatr Soc 2005;53:695-9.

45. Neufeld KJ, Hayat MJ, Coughlin JM et al. Evaluation of two intensive care delirium screening tools for non-critically ill hospitalized patients. Psychosomatics 2011;52:133-40.

46. McNicoll L, Pisani MA, Ely EW, Gifford D, Inouye SK. Detection of delirium in the intensive care unit: comparison of confusion assessment method for the intensive care unit with confusion assessment method ratings. J Am Geriatr Soc 2005;53:495-500.

47. Wang C, Wu Y, Yue P et al. Delirium assessment using Confusion

Assessment Method for the Intensive Care Unit in Chinese critically ill patients. J Crit Care 2013;28:223-9.

48. Sessler CN, Gosnell MS, Grap MJ et al. The Richmond Agitation-Sedation Scale: validity and reliability in adult intensive care unit patients. Am J Respir Crit Care Med 2002;166:1338-44.

49. Ely EW, Truman B, Shintani A et al. Monitoring sedation status over time in ICU patients: reliability and validity of the Richmond Agitation-Sedation Scale (RASS). Jama 2003;289:2983-91.

50. Ely EW, Inouye SK, Bernard GR et al. Delirium in mechanically ventilated patients: validity and reliability of the confusion assessment method for the intensive care unit (CAM-ICU). Jama 2001;286:2703-10.

51. Spitzer RL, Kroenke K, Williams JB, Lowe B. A brief measure for assessing generalized anxiety disorder: the GAD-7. Archives of internal medicine 2006;166:1092-7.

52. Bastien CH, Vallieres A, Morin CM. Validation of the Insomnia Severity Index as an outcome measure for insomnia research. Sleep medicine 2001;2:297-307.

53. Carlsson AM. Assessment of chronic pain. I. Aspects of the reliability and validity of the visual analogue scale. Pain 1983;16:87-101.

54. Peterson JF, Pun BT, Dittus RS et al. Delirium and its motoric subtypes: a study of 614 critically ill patients. J Am Geriatr Soc 2006;54:479-84.

55. Alshurafa M, Briel M, Akl EA et al. Inconsistent definitions for intention-to-treat in relation to missing outcome data: systematic review of the methods literature. PLoS One 2012;7:e49163.

56. Li H, Han D, Hou Y, Chen H, Chen Z. Statistical inference methods for two crossing survival curves: a comparison of methods. PLoS One 2015;10:e0116774.

57. Liu K, Qiu P, Sheng J. Comparing two crossing hazard rates by Cox proportional hazards modelling. Stat Med 2007;26:375-91.

58. van der Kooi AW, Zaal IJ, Klijn FA et al. Delirium detection using EEG: what and how to measure. Chest 2015;147:94-101.

59. Fritz BA, Kalarickal PL, Maybrier HR et al. Intraoperative Electroencephalogram Suppression Predicts Postoperative Delirium.

Anesth Analg 2016;122:234-42.

60. Hesse S, Kreuzer M, Hight D et al. Association of electroencephalogram trajectories during emergence from anaesthesia with delirium in the postanaesthesia care unit: an early sign of postoperative complications. Br J Anaesth 2019;122:622-34.

61. Schaefer ST, Koenigsperger S, Olotu C, Saller T. Biomarkers and postoperative cognitive function: could it be that easy? Curr Opin Anaesthesiol 2019;32:92-100.

62. Leung JM, Sands LP, Mullen EA, Wang Y, Vaurio L. Are preoperative depressive symptoms associated with postoperative delirium in geriatric surgical patients? J Gerontol A Biol Sci Med Sci 2005;60:1563-8.

63. Wu X, Sun W, Tan M. Incidence and Risk Factors for Postoperative Delirium in Patients Undergoing Spine Surgery: A Systematic Review and Meta-Analysis. Biomed Res Int 2019;2019:2139834.

64. Ghoneim MM, O'Hara MW. Depression and postoperative complications: an overview. BMC Surg 2016;16:5.

65. Alattas SA, Smith T, Bhatti M, Wilson-Nunn D, Donell S. Greater pre-operative anxiety, pain and poorer function predict a worse outcome of a total knee arthroplasty. Knee Surg Sports Traumatol Arthrosc 2017;25:3403-10.

66. Wang JP, Lu SF, Guo LN, Ren CG, Zhang ZW. Poor preoperative sleep quality is a risk factor for severe postoperative pain after breast cancer surgery: A prospective cohort study. Medicine (Baltimore) 2019;98:e17708.
